# SPM Differences in Gait Pattern of Women After Total Hip Replacement: A Longitudinal Study

**DOI:** 10.3390/jcm14124316

**Published:** 2025-06-17

**Authors:** Krzysztof Aleksandrowicz, Wojciech Kosowski, Agata Michalska, Sławomir Winiarski

**Affiliations:** 1 Faculty of Physiotherapy, University Center for Physiotherapy and Rehabilitation, Wroclaw Medical University, Chalubińskiego 3, 50-368 Wroclaw, Poland; krzysztof.aleksandrowicz@umw.edu.pl; 2 Institute of Heart Diseases, University Hospital, Borowska 213, 50-556 Wroclaw, Poland; wojciech.kosowski@umw.edu.pl; 3 Institute of Heart Diseases, Wroclaw Medical University, Borowska 213, 50-556 Wroclaw, Poland; 4 Faculty of Health Sciences, Collegium Medicum of Jan Kochanowski, University in Kielce, 25-317 Kielce, Poland; michalska.agata@ujk.edu.pl; 5 Department of Physiology and Biomechanics, Wroclaw University of Health and Sport Sciences, 51-612 Wroclaw, Poland

**Keywords:** total hip replacement, gait analysis, rehabilitation, kinematic changes, postoperative recovery, motion analysis

## Abstract

**Background**: Total Hip Replacement (THR) is a standard treatment for advanced hip osteoarthritis; yet, its effects on gait recovery remain understudied. This study examines gait pattern changes in women undergoing monitored rehabilitation after unilateral THR, using Statistical Parametric Mapping (SPM) to detect significant motion differences over time. **Methods**: This longitudinal study included 32 women who underwent primary cementless THR. Gait was assessed preoperatively and postoperatively at 6 weeks, 3 months, 6 months, and 12 months using a motion analysis system. Repeated measures ANOVA and post hoc SPM{t} analyses were conducted to evaluate significant gait changes across time points. **Results**: Significant improvements (*p* < 0.05) were observed in spatio-temporal parameters. Velocity increased from 0.42 ± 0.10 m/s (Ex1) to 0.72 ± 0.06 m/s (Ex5), stride length from 0.85 ± 0.12 m to 1.15 ± 0.07 m, and step length (involved leg) from 0.32 ± 0.08 m to 0.48 ± 0.05 m. Cycle time decreased from 1.50 ± 0.20 s to 1.22 ± 0.10 s, indicating improved gait efficiency. Post hoc SPM{t} analysis revealed significant kinematic changes in hip flexion-extension, knee flexion, and pelvic tilt, particularly between Ex2 and Ex3. Statistically significant improvements (*p* < 0.001) were observed in key spatio-temporal parameters. **Conclusions**: Gait parameters improved significantly within the first year post-THR, with the most pronounced changes occurring between the early and mid-term recovery phases. These findings support the need for targeted rehabilitation strategies in the first six months post-surgery. SPM analysis provides a robust method for detecting subtle gait adaptations, contributing to the refinement of post-THR rehabilitation strategies.

## 1. Introduction

The World Health Organization (WHO) has identified osteoarthritis (OA) as a significant disease of modern times, particularly highlighting its prominence in the transition from the 20th to the 21st century. According to the WHO, in 2019, about 528 million people worldwide were living with OA, an increase of 113% since 1990 [1,2]. The knee is the most frequently affected joint, followed by the hip and the hand. With ageing populations and increasing rates of obesity and injury, the incidence of OA is anticipated to escalate further [3,4]. This condition presents significant medical dilemmas and imposes substantial social and economic burdens. Specifically, in Poland, the impact of OA is profound, with an estimated eight million individuals affected by the disease, nearly three million of whom endure hip-related afflictions [5].

In response to the growing challenge of OA, particularly concerning hip lesions, Total Hip Replacement (THR) has been innovatively developed as a pivotal orthopaedic solution [6]. Designed to mitigate pain and enhance the functional mobility of those grappling with severe hip joint disorders, THR involves the surgical replacement of the afflicted hip joint with prosthetic components [6,7]. This procedure aspires to reinstate the integrity and functionality of the hip joint, offering a renewed lease on mobility and quality of life for patients [8]. Recent research in gait analysis post-THR has shed light on various facets of rehabilitation outcomes and gait asymmetry, offering valuable insights into the dynamics of patient recovery. A narrative review by Bahadori et al. [9] underscores the utility of gait analysis as a tool for evaluating walking ability post-THR and assessing long-term physical performance outcomes relative to the general population. Furthermore, studies [10,11] reveal that while gait improvements post-THR are observable, significant deviations from healthy controls persist up to a year post-surgery, particularly in patients with poor preoperative gait patterns [10]. Additionally, Pagano et al. [12] have highlighted the significance of modern technologies for gait analysis in rehabilitation, demonstrating substantial improvements in mobility and symmetry of spatiotemporal parameters within a month post-THR [12].

In the context of post-THR rehabilitation, the utilisation of advanced gait analysis techniques, such as video analysis, has been shown to improve outcomes significantly. For instance, recent studies have highlighted the benefits of interventions focusing on enhancing step length symmetry and the use of larger femoral heads in prostheses to achieve better gait patterns, underscoring the potential of gait analysis-driven strategies in enhancing post-operative recovery [12,13]. Gait analysis serves as a critical tool in the identification, quantification, and assessment of gait asymmetries and deviations, which are pivotal in tailoring rehabilitation programs to meet the individual needs of patients post-THR [7,11,14]. The significance of gait analysis extends beyond mere identification; it allows for objectively evaluating rehabilitation progress over time. By quantitatively assessing changes in gait parameters, clinicians can monitor the effectiveness of rehabilitation strategies, make informed decisions about modifications to treatment plans, and set realistic, achievable goals for patients [15,16]. This ongoing assessment ensures that rehabilitation programs remain dynamic and responsive to the evolving needs of patients, thereby optimising functional outcomes [17]. Moreover, gait analysis facilitates the personalisation of rehabilitation programs [16]. This personalised approach not only enhances the efficiency of rehabilitation but also contributes to greater patient engagement and adherence to prescribed therapeutic activities [17].

Gait asymmetry, a common occurrence post-THR, can significantly impact the functional recovery and overall quality of life of patients. Asymmetries in gait patterns may lead to compensatory strategies that, while adaptive in the short term, could result in long-term musculoskeletal issues, including joint stress, pain, and a heightened risk of falls [18,19]. Through the detailed insights provided by gait analysis, healthcare professionals can identify specific deviations in gait patterns, such as limping, unequal stride lengths, and alterations in weight distribution, which are crucial for the development of targeted rehabilitation interventions [20]. SPM’s ability to analyse gait and movement patterns across the entire cycle offers a nuanced understanding of movement asymmetry, with significant implications for diagnosis, treatment, and rehabilitation in physical therapy [21,22,23]. The growing body of research utilising SPM in biomechanics and physical therapy underscores its pivotal role in advancing our understanding of complex gait dynamics, marking a significant step forward in the quest for precision in movement analysis. To be precise, Statistical Parametric Mapping (SPM) represents a significant advancement in the field of biomechanics, particularly in the analysis of complex gait data. This advanced statistical tool is instrumental in detecting subtle changes and differences in motion patterns, offering a sophisticated approach to understanding the nuances of human movement. SPM operates by creating spatially extended statistical processes, allowing for the analysis of data across the entire gait cycle, rather than at discrete points, thus providing a comprehensive view of gait dynamics.

The relevance of SPM in biomechanics and physical therapy is profound, particularly in the context of gait analysis. Traditional gait analysis methods, which often rely on discrete data points or averages, may overlook subtle but clinically significant variations in gait patterns. SPM, however, facilitates a detailed examination of these patterns over time, enabling the detection of asymmetries or deviations that might not be apparent through conventional analysis. This capability makes SPM an invaluable tool for researchers and clinicians aiming to assess the effectiveness of interventions, understand the biomechanical changes associated with pathological conditions, or monitor rehabilitation progress. Recent studies employing SPM in biomechanics have underscored its effectiveness in analysing complex gait data across various populations. For instance, Mestanza Mattos et al. [24] utilised SPM alongside the Gait Profile Score to describe walking alterations in individuals with multiple sclerosis, highlighting the complementary strengths of SPM in capturing the full spectrum of gait changes [24]. Similarly, a scoping review by Yona et al. [25] summarised the application of one-dimensional SPM in sports biomechanics, revealing its potential to bridge research gaps in the kinetic and kinematic analysis of lower limb joints. These studies exemplify the broad applicability and precision of SPM in gait analysis, from diagnosing movement disorders to enhancing athletic performance. Moreover, the versatility of SPM extends beyond traditional gait analysis to innovative applications in clinical movement analysis. Alhossary et al. [26] demonstrated how SPM provides a quantitative and coherent assessment of joint dynamics, overcoming the subjectivity errors inherent in other methods [26]. This approach enhances the accuracy of biomechanical assessments and contributes to the development of targeted rehabilitation protocols.

The necessity for longitudinal gait analysis, with a particular focus on symmetry, is undeniably crucial. Understanding the dynamics of gait changes over different post-operative periods enhances the ability to tailor rehabilitation protocols effectively, thereby fostering optimal recovery trajectories for THR patients. This study aims to bridge the existing gap in knowledge, providing a foundation for future research endeavours and clinical practices aimed at improving patient outcomes post-THR with a focus on unilateral hip replacements and employing SPM for in-depth analysis. Studies such as that by van Drongelen et al. [10] highlight persistent deviations from physiological gait patterns up to a year post-surgery, illuminating the prolonged trajectory of gait recovery and the necessity for ongoing evaluation. This protracted recovery process accentuates the importance of understanding gait dynamics across different post-operative periods. A longitudinal analysis enables clinicians to chart the progression of recovery, offering a granular understanding of how and when gait asymmetry diminishes, thereby refining rehabilitation protocols to be more responsive to the evolving needs of patients. Moreover, the current body of literature calls for further investigation into the long-term effects of THR on gait dynamics, with a particular focus on developing targeted rehabilitation programs that can address these identified gaps and enhance patient outcomes effectively.

The primary objective of this study is to elucidate the dynamics and significance of changes in the gait pattern between successive research periods in women undergoing a monitored rehabilitation process following unilateral Total Hip Replacement (THR). This study aims to determine when these changes are most pronounced and which specific movements are affected, using a post hoc analysis to refine rehabilitation strategies and enhance recovery outcomes. Specifically, the research seeks to answer the following questions:

(1) Between which research periods are the most significant changes in gait patterns observed for the involved and uninvolved limbs?
(2) What specific movements and joint kinematics exhibit the most significant variations over time during the monitored rehabilitation process?
(3) How do changes in kinematic and kinetic gait parameters evolve across different phases of rehabilitation (preoperative, early postoperative, mid-term, and long-term recovery)?
(4) What are the clinical implications of observed gait changes regarding rehabilitation effectiveness and functional recovery?
(5) How does applying Statistical Parametric Mapping (SPM) contribute to identifying and quantifying significant gait pattern differences post-THR?

To adequately address these inquiries, the study leverages SPM to analyse gait patterns comprehensively and statistically robustly.

## 2. Materials and Methods

This was a longitudinal observational study designed to track changes in gait patterns over time.

### 2.1. Participants, Inclusion Criteria

The study included 32 female patients diagnosed with unilateral hip osteoarthritis and qualified for primary cementless total hip arthroplasty as a treatment for advanced degenerative changes in the hip joint. The group had the following characteristics (mean ± standard deviation): age: 65.34 ± 9.40 years; body mass: 76.10 ± 16.42 kg; height: 158.03 ± 6.03 cm; and BMI: 30.40 ± 6.35 kg/m^2^. All participants were selected using a purposive sampling method, operated on, and assessed at different rehabilitation stages by a team of orthopaedic specialists from the Specialist Rehabilitation–Orthopaedic Centre in Wrocław, Poland. Patients included in the study did not report pain or range-of-motion limitations in the adjacent hip joint, other lower limb joints, or the lumbar-sacral spine, which was confirmed through radiological and physical examinations by the qualifying physician. The surgical procedure was performed in a lateral position using a posterior-lateral approach.

Exclusion criteria encompassed patients with comorbid musculoskeletal disorders affecting gait, prior lower limb surgeries, neurological conditions influencing movement patterns, severe systemic diseases that could impair recovery, and those who had undergone bilateral THR before the study commenced. Individuals who reported pain or functional limitations in the contralateral limb or lumbar spine were excluded. Participants were discharged from the hospital on the seventh postoperative day after undergoing initial rehabilitation and gait training with elbow crutches. They were instructed on proper ambulation on flat surfaces and stairs with partial weight-bearing on the operated limb. Each participant provided written informed consent to participate in the study and was informed of their right to withdraw at any stage. To minimise sex-based variability, this study focused exclusively on female patients (from one female orthopaedic ward).

Gait analysis was conducted at five intervals: Examination 1: One or two days before the surgical procedure. Examination 2: Six weeks post-operation. Examination 3: Three months post-operation. Examination 4: Six months post-operation. Examination 5: Twelve months post-operation.

To calculate the desired sample size, the G*Power calculator (version 3.1.9.7 for Windows) was used to ensure adequate statistical power for detecting significant differences in gait parameters across research periods. The sample size estimation (25 minimum) was based on an effect size of 0.5 (medium effect), a statistical power of 0.80, an alpha level of 0.05, and repeated measures ANOVA as the chosen statistical test.

### 2.2. Measurement Set-Up

The research was performed in an ISO certified laboratory for biomechanical analysis. The research received a positive opinion from the ethics committee within the research project KB-12/2011 framework. The kinematic and kinetic parameters of gait were measured using the film method with the BTS Smart E optoelectronic motion analysis system equipped with BTS Smart Analyzer^®^ 1.1 software (BTS Bioengineering, Milan, Italy). The film method is considered a gold standard among motion analysis systems due to its high precision [27,28]. The system included 6 digital cameras operating in the infrared range (1.1 μm infrared light with 120 fps in 768 × 576 px resolution).

### 2.3. Measurement Procedure and Calculations

Following a modified version of the Helen Hayes–Davis model tailored for the lower limbs, twenty-six photo reflective markers were affixed to the participant’s body. These markers were positioned on the skin following the International Society of Biomechanics (ISB) guidelines [29,30,31], ensuring symmetry between the left and right sides. The markers denoted specific anatomical sites: the C7 vertebra, both acromion processes, two markers on the Th2 spinous process, two on the Th10 spinous process, two over the xiphoid process, the suprasternal notch, the sacrum, both anterior superior iliac spines (ASIS), both greater trochanters, two on the thighs (located midway between the greater trochanter and the lateral condyle of the femur), two at the knees (spanning the lateral condyle of the femur to the fibula), two on the shanks (midway between the head of the fibula and the lateral malleolus), both lateral malleoli, both second metatarsal heads, and two on the heels (which were removed following static calibration).

The participant was instructed to walk approximately 6 m at a self-selected speed, enabling the capture of 2–4 complete gait cycles (depending on walking speed) in a single pass. Four repetitions of this walking task were recorded, resulting in 8 to 16 gait cycles being documented for the specific research period. Regarding camera calibration and 3D reconstruction, raw measurement data detailing marker positions over time were converted into joint angles. The angles of particular interest in this study included the following:

- Pelvic tilt (PTILT) involves the pelvis tilting forwards (indicating a positive tilt) or backwards (indicating a negative tilt) in the sagittal plane, which occurs due to its rotation around the mediolateral axis.
- Pelvic obliquity (POBLI) is characterised by the pelvis moving upwards (positive obliquity) or downwards (negative obliquity) relative to a global coordinate system in the coronal plane, resulting from the mediolateral axis’s rotation out of alignment with the horizontal plane.
- Pelvic rotation (PROT) refers to the inward (positive) or outward (negative) rotation of the pelvis in the transversal plane, which is due to the mediolateral axis rotating around the vertical axis.
- Hip flexion-extension (HPFE) denotes the forward (flexion, positive) or backward (extension, negative) movement of the thigh bone (femur) in the sagittal plane, as a result of its rotation around the mediolateral axis at the hip joint.
- Hip abduction-adduction (HPAA) describes the movement of the femur away from (abduction, positive) or towards (adduction, negative) the midline of the body in the coronal plane, occurring through the rotation of the proximal–distal axis away from the sagittal plane.
- Hip rotation (HPROT) involves the internal (positive) or external (negative) rotation of the femur in the transversal plane, which happens due to rotation about the proximal–distal axis.
- Knee flexion-extension (KFE) is the bending (flexion, positive) or straightening (extension, negative) movement of the lower leg (tibia) in relation to the femur in the sagittal plane, caused by the rotation of the proximal–distal axis around the mediolateral axis.
- Knee varus/valgus indicates the movement of the lower leg (tibia) towards (varus, positive) or away from (valgus, negative) the midline of the body in the coronal plane.
- Ankle dorsiflexion-plantarflexion (AFE) represents the upward (dorsiflexion, positive) or downward (plantarflexion, negative) movement of the foot in relation to the lower leg (tibia) in the sagittal plane.

The report containing the angles was arranged according to the guidelines for reporting motion analysis results [32] in such a way that in the columns of the report from left to right are the planes of observation (sagittal, frontal, transversal planes) and in the rows from top to bottom are the levels of observation (pelvis, hip, knee, ankle).

The stance/swing phases were identified based on the vertical movement of the heel or foot marker. Initial contact (foot strike) is the instance when the heel marker reaches the minimum height, and the swing begins when the foot marker starts ascending (foot is off). In the BTS Smart Analyzer^®^ software, markers are placed semiautomatically on the reported resulting graph. All reported graphs include mean value and ±1 standard deviation from the mean as a function of normalised cycle time. Markers are placed, and reports are created by an experienced laboratory technician.

### 2.4. Statistical Calculations

Repeated measures ANOVA was used to assess changes in spatio-temporal gait parameters across five examination periods (Ex1–Ex5). The analysis evaluated velocity, stride length, step length (on the involved leg), and cycle time to determine significant within-subject effects over time. F-values and *p*-values were reported to indicate statistical significance, with *p* < 0.05 considered significant.

**Figure 1:**
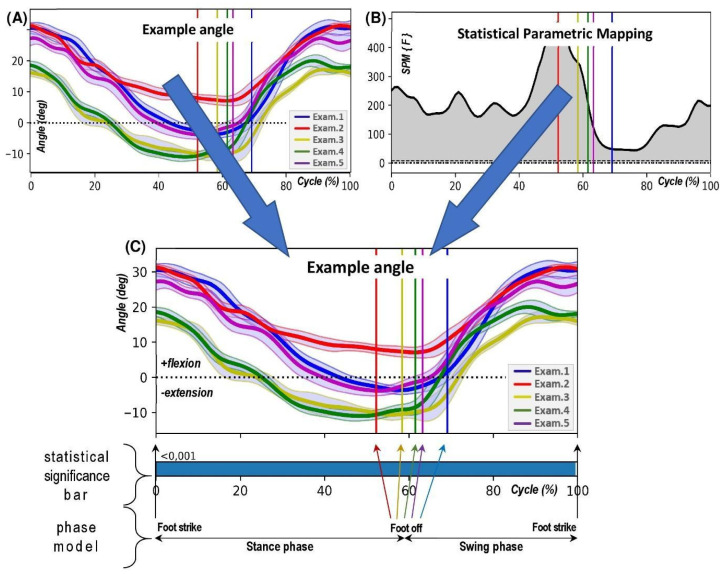
The methodology employed to generate a result chart for an example angle. Initially, the original graph presents the mean angle values, along with the standard deviation, for successive research periods from Exam.1 to Exam.5 (**Panel A**). This data is then subjected to a statistical process. The outcome of Statistical Parametric Mapping (SPM) computations is depicted in a chart that exhibits the statistical function’s value (**Panel B**). In the final step (**Panel C**), statistical information is superimposed onto the angle graph as a statistical significance bar. The resulting chart also includes details about the gait phases. The length of the stance phase (from foot strike to foot off) differs across the research periods and is indicated by vertical, coloured lines. Each line’s colour corresponds to the angle trajectory’s colour for that specific research period, as described in the legend.

In addition, the Python 3.12.6 package for 1-dimensional Statistical Parametric Mapping (SPM1D) was used to evaluate the statistical information between subsequent examination periods for each angular data (Figure 1A) [23,33]. SPM was chosen over traditional discrete approaches as it enables statistical analysis of entire time-series waveforms, providing greater sensitivity to temporal patterns and subtle differences in joint kinematics that single-point or peak-value comparisons may not capture. Explicitly, an SPM-ANOVA with repeated measures was performed to investigate the overall trend of movement kinematic changes as Pataky et al. described [34]. In addition, a post hoc one-sample t test was performed between successive test periods to answer the specific question. The SPM conducts statistical testing at the continuum level (for time series) in a conceptually simple manner. For 1D datasets, it quantifies the probability that smooth, random 1D continua would produce a test statistic continuum whose maximum exceeds a defined test statistic value (t or F value) [21]. The test statistics (thick black line in Figure 1B) depicted the test statistic continuum, which in this case is an F-continuum (SPM{F}). From a classical hypothesis testing perspective, the null hypothesis was rejected at alpha = 0.05 if the SPM{F} exceeded the alpha threshold. The calculated probability (*p*) value indicates the probability that smooth, random continua would produce a supra-threshold cluster as broad as the observed cluster. Here, “smooth” means the same smoothness as the residual continua, and “broad” means the proportion of the continuum spanned by a suprathreshold cluster. To save some space and simplify the calculations, the statistical information is combined and transferred into a horizontal bar representation and placed under the angle graph (Figure 1C). The Mean angle is visualised along with ±1 standard deviation (SD) clouds around the mean value.

A post hoc analysis was performed using the 1D SPM *t*-test (SPM{t}) to determine specific gait pattern differences between successive examination periods. Statistical significance was determined using a critical threshold based on random field theory, with an alpha level set at 0.05. To account for multiple comparisons, a Bonferroni correction was applied, adjusting the critical *p*-value accordingly [35].

## 3. Results

Basic spatio-temporal parameters depicting the average gait of the experimental group are presented in Table 1.

The brief analysis of spatio-temporal gait parameters revealed significant improvement trends across the monitored rehabilitation periods (*p* < 0.05 for all parameters). Velocity increased progressively from 0.42 ± 0.10 m/s preoperatively (Ex1) to 0.72 ± 0.06 m/s at 12 months postoperatively (Ex5), with a highly significant effect (F(4,124) = 177.71, *p* < 0.001). Stride length followed a similar pattern, increasing from 0.85 ± 0.12 m in Ex1 to 1.15 ± 0.07 m in Ex5, reflecting enhanced step dynamics and gait efficiency (F(4,124) = 195.54, *p* < 0.001). Step length on the involved leg also exhibited significant growth (F(4,124) = 61.56, *p* < 0.001), progressing from 0.32 ± 0.08 m preoperatively to 0.48 ± 0.05 m at Ex5, indicating an increased ability to bear weight and advance the limb symmetrically. Cycle time showed a significant reduction over time (F(4,124) = 141.73, *p* < 0.001), decreasing from 1.50 ± 0.20 s in Ex1 to 1.22 ± 0.10 s in Ex5, suggesting improved cadence and step regulation. These results confirm a statistically significant and clinically meaningful improvement in gait parameters over the one-year rehabilitation period. The progressive increases in velocity, stride length, and step length, combined with a decrease in cycle time, indicate a restoration of dynamic gait stability and efficiency post-THR.

**Figure 2:**
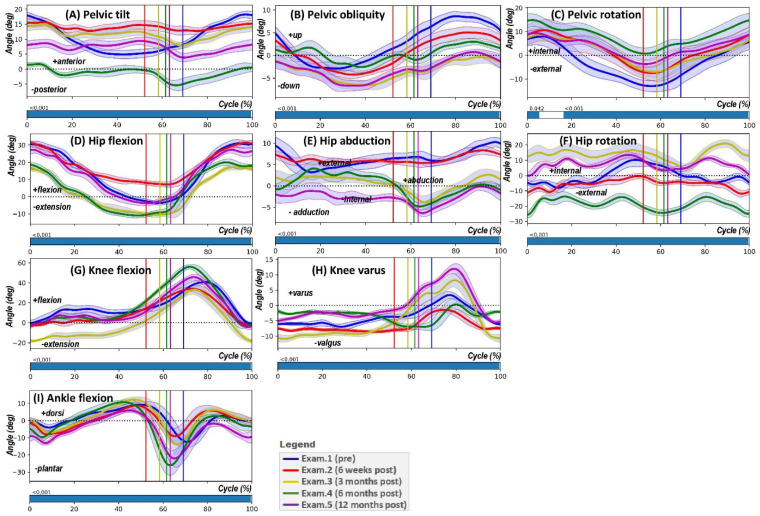
Sequential analysis of gait cycle kinematics through SPM{F}. This figure illustrates the Statistical Parametric Mapping (ANOVA with repeated measures—SPM{F}) results for pelvic, hip, knee, and ankle angles across four examination intervals. Each subfigure (**A**–**I**) represents the mean angle trajectory for PTILT, POBLI, PROT, HPFE, HPAA, HPROT, KFE, Knee varus/valgus, and AFE, respectively. Vertical coloured lines denote statistically significant deviations within the gait cycle, corresponding to pre-operation (Exam.1), and post-operation phases at 6 weeks (Exam.2), 3 months (Exam.3), 6 months (Exam.4), and 12 months (Exam.5). Areas above the statistical threshold in the SPM{F} charts highlight phases of the gait cycle with significant kinematic changes, contributing to a comprehensive understanding of the rehabilitation process.

The results of the significance analysis of differences in angular kinematics between successive measurement periods are depicted in Figure 2. The horizontal bars in each subfigure denote the specific points within the gait cycle where the SPM{F} test statistics cross the threshold for significance, illustrating the comparative overall differences in joint kinematics between the various examination periods.

The general results of the SPM analysis (ANOVA with repeated measures—SPM{F}) for the specified angles (Figure 2) can be interpreted as follows: variations in pelvic tilt throughout the gait cycle are evident, with statistically significant differences occurring at specific points. Notably, a prominent divergence arises around the initial contact and mid-stance phases. Pelvic obliquity displays fluctuations with discernible significant changes during the cycle, suggesting alterations in the asymmetry of the pelvis during different gait phases. Changes in pelvic rotation are marked, with statistical significance particularly observable in the transitions between the stance and swing phases, indicating alterations in the rotational movement of the pelvis.

Hip flexion and extension show a pattern of significant change, especially around the points of foot strike and foot off, reflecting the dynamic changes in hip angle during the gait cycle. There are notable periods within the gait cycle where hip abduction and adduction demonstrate significant differences, potentially highlighting variations in the lateral movement of the hip through successive examinations. The hip rotation analysis reveals statistically significant shifts at particular phases of the gait cycle, indicating changes in internal and external rotation of the hip across the examined periods.

The knee’s flexion and extension phases display statistically significant differences, with substantial changes occurring predominantly around the stance phase, illustrating variations in the knee’s movement as it transitions from bending to straightening. The analysis of knee varus and valgus indicates statistically significant differences at various stages of the gait cycle, which may reflect changes in the medial and lateral alignment of the knee.

Ankle movement between dorsiflexion and plantarflexion shows significant variation, particularly at the junctures of the stance-to-swing transition, pointing to changes in how the ankle adapts and contributes to the gait cycle.

SPM{F} graphs accompanying each angle visually represent where these significant changes occur within the gait cycle, characterised by the regions where the SPM{F} line crosses the statistical threshold, indicating where the variance amongst the different examination periods is significant.

**Figure 3:**
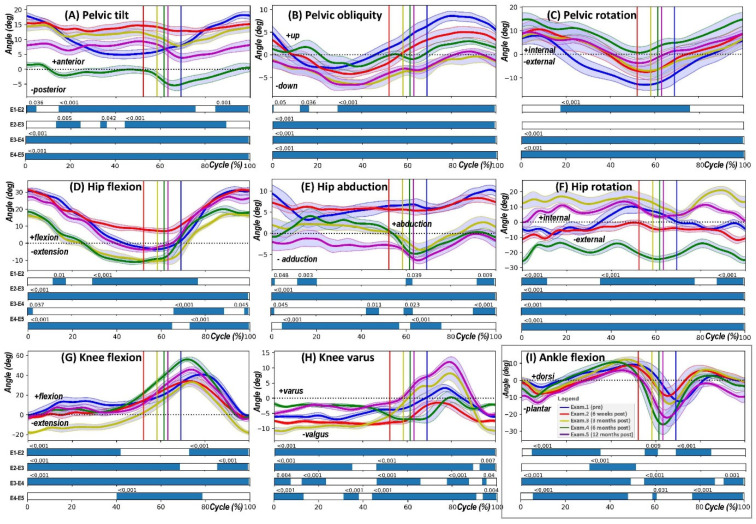
Pair-wise SPM{t} test analysis of joint angle variations across gait cycles. This figure demonstrates the temporal patterns of significant differences in joint angles between consecutive examination periods, as identified by the SPM{t} statistical test. Subfigures (**A**–**I**) present pair-wise comparisons for PTILT, POBLI, PROT, HPFE, HPAA, HPROT, KFE, Knee varus/valgus, and AFE angles, respectively. The horizontal bars below each graph denote the statistical significance of differences, with specific *p*-values indicating the significance level at corresponding percentages of the gait cycle. These results elucidate the biomechanical evolution of joint kinematics following clinical intervention, captured at preoperative (Exam.1), and postoperative phases at 6 weeks (Exam.2), 3 months (Exam.3), 6 months (Exam.4), and 12 months (Exam.5). Areas above the statistical threshold in the SPM{F} charts highlight phases of the gait cycle with significant kinematic changes, contributing to a comprehensive understanding of the rehabilitation process.

Specifically, the results of the post hoc pair-wise SPM{t} test analysis of joint angle variations across gait cycles are presented in Figure 3, illustrating the temporal patterns of significant differences in joint angles between consecutive examination periods.

The SPM{t} analysis for the defined angles across consecutive examination periods—preoperative (Exam.1), 6 weeks postoperative (Exam.2), 3 months postoperatively (Exam.3), 6 months postoperatively (Exam.4), and 12 months postoperatively (Exam.5)—revealed significant alterations in joint kinematics throughout the gait cycle. Explicitly, significant changes in pelvic tilt were observed around 60% of the gait cycle, where the SPM{t} test indicated a *p*-value < 0.001. Pelvic obliquity demonstrated notable differences primarily in the mid-cycle phase, underscored by significant *p*-values (<0.001). Changes in pelvic rotation were identified at around 60% and 80% of the gait cycle, suggesting substantial modifications in postural control following THR.

For the hip joint, statistically significant differences in flexion-extension were evident, particularly during the initial and terminal stance phases, where the SPM{t} test consistently indicated *p*-values < 0.001. Hip abduction–adduction also exhibited significant variations, especially during the transition from late stance to early swing, reinforcing the importance of lateral stability post-surgery. Additionally, hip rotation demonstrated significant alterations, with *p*-values < 0.001 at multiple gait cycle intervals, highlighting functional adaptations in the transverse plane.

For the knee and ankle kinematics, changes in knee flexion-extension were statistically significant during mid-stance and pre-swing phases, with SPM{t} results showing *p*-values < 0.001, reflecting enhanced dynamic knee control during weight transfer. Knee varus/valgus variations also reached statistical significance across multiple cycle points, suggesting postural adaptations over time. In the ankle, dorsiflexion-plantarflexion changes were prominent at around 50% of the gait cycle, where SPM{t} results indicated significant differences (*p* < 0.001), reflecting neuromuscular recovery during push-off and foot clearance.

Overall, the pair-wise SPM{t} comparisons presented in Figure 3 illustrate the most pronounced biomechanical alterations in the pelvic, hip, knee, and ankle joints at different stages of the rehabilitation process. The figure’s horizontal bars mark these differences’ statistical significance, emphasising critical phases of post-THR gait adaptation.

## 4. Discussion

This study demonstrated significant improvements in spatio-temporal gait parameters and joint kinematics over a 12-month rehabilitation period following unilateral THR in women. The most pronounced changes occurred between 6 weeks and 6 months postoperatively, particularly in hip, knee, and pelvic motion, as revealed by SPM analysis. The findings of this study provide crucial insights into the temporal evolution of gait patterns in women undergoing monitored rehabilitation following unilateral THR. In particular, the SPM analysis revealed distinctive patterns that hadn’t been observed before. Significant changes in spatio-temporal and joint kinematics were observed at various phases of the rehabilitation process, highlighting the progressive adaptation of movement strategies as patients recover.

In response to the first research question, the most substantial changes in gait parameters were observed between Examination 2 (6 weeks) and Examination 4 (6 months), aligning with the period of increased rehabilitation intensity and patient mobility. This aligns with findings by Agostini et al. [36], who similarly observed significant improvements in stride length, cadence, and walking speed during this period, supported by EMG data indicating more normalised muscle activation. Similarly, Horstmann et al. [37] noted substantial gains in walking speed and step length between 6 weeks and 6 months post-THR, attributed to improved weight-bearing and increased rehabilitation engagement. This aligns with recent findings from Ramadanov et al. [38], who demonstrated in two large-scale meta-analyses that minimally invasive THR approaches, such as the direct anterior approach, significantly improve early postoperative functional outcomes, including walking speed and symmetry. Patterson et al. [39] confirmed that gait symmetry improvements were most prominent during the first 3–6 months of rehabilitation, even in other patient groups, emphasising the importance of this phase. Wada et al. [40] reported that gait stability significantly increased between 6 weeks and 6 months in women with hip osteoarthritis, suggesting effective neuromuscular recalibration. These findings collectively reinforce that this phase is a pivotal window for enhancing spatio-temporal gait dynamics. As noted, all spatio-temporal parameters significantly improved during this interval, indicating a critical window for functional restoration.

Regarding the second research question, movements such as hip and knee flexion-extension and pelvic tilt and obliquity exhibited the most significant variability. The SPM{t} analyses demonstrated that these changes were not uniformly distributed across the gait cycle but concentrated at critical functional phases, particularly during foot strike, mid-stance, and toe-off. For instance, pelvic tilt showed significant changes around 60% of the gait cycle, while pelvic obliquity varied most notably during mid-cycle transitions. Hip flexion-extension revealed marked differences at both initial contact and terminal stance, indicating the role of muscle control during loading and push-off. The knee exhibited variability in flexion-extension primarily during mid-stance and pre-swing, highlighting dynamic knee adaptation under loading and unloading. These fluctuations may reflect the ongoing neuromuscular recalibration occurring throughout rehabilitation, and their magnitude and timing suggest key targets for phase-specific physiotherapeutic intervention. These results align with findings by Pincheira et al. [41], who observed hip kinematic variability during 11–43% of the gait cycle using SPM, particularly at mid-stance and toe-off. Rao et al. [42] in females with hip pain also demonstrated altered pelvic kinematics and elevated anterior pelvic tilt across key peaks using full-cycle SPM analysis. Loppini et al. [43] specifically identified deviations in pelvic motion during heel-strike and toe-off in unilateral and bilateral THA. Alves et al. [44] demonstrated that weight-bearing symmetry and ipsilateral loading improvements post-THA are activity-dependent, with persistent deficits observed during walking up to 12 weeks postoperatively, despite improvements in other activities such as standing and sit-to-stand transitions. Ryan et al. [45] reported pronounced hip restriction at initial contact and terminal stance and showed extensive hip flexion-extension variability during loading and push-off. Wesseling et al. [46] and Vasiljeva et al. [47], using SPM, further confirmed dynamic kinematic adaptations at mid-stance and toe-off. Collectively, these studies reinforce that the variability identified through SPM in the present study is not only phase-specific but also consistent with existing literature on post-THR gait adaptation.

For the third research question, no significant changes were expected between the preoperative examination and the surgery itself, as the gap between Examination 1 and the surgery was minimal (1–2 days). However, slight but statistically significant improvements were observed in the early postoperative phase (Ex1–Ex2) despite factors like limited weight-bearing, wound healing, and early-stage pain. This is consistent with findings from Zhang & Xiao [48], who reported in a meta-analysis that early functional improvements, even under load restrictions, were amplified by proprioceptive and neuromuscular therapies. Mumbleau & Schilaty [49] also observed early neural adaptations post-hip surgery, including improvements in muscle recruitment despite clinical limitations. The most dynamic phase of recovery was clearly identified between Ex2 and Ex4, reflecting the benefits of progressive load-bearing and neuromuscular adaptation following physician clearance. Jin et al. [50] highlighted that functional benefits become more pronounced after the initial postoperative healing period, while Hjorth et al. [51] and Adler et al. [52] noted that muscle power restoration and neuromuscular coordination improve substantially during mid-term recovery. Altogether, this confirms that the earliest phase shows only modest gains.

Addressing the fourth research question, these findings support the implementation of intensive, phase-specific rehabilitation starting as early as 2–3 weeks post-surgery, once clinical clearance has been obtained. Between weeks 3 and month 4, patients should undergo structured gait training focused on increasing walking speed, stride length, and reducing cycle time—key markers of neuromuscular improvement observed in this period (e.g., velocity from 0.42 to 0.72 m/s; stride length from 0.85 to 1.15 m; cycle time from 1.50 to 1.22 s). Clinicians are advised to incorporate load-bearing progression, dynamic balance exercises, and task-specific gait drills that emphasise stance and pre-swing phases, where the most pronounced joint kinematic changes occur. Rehabilitation protocols should prioritise strengthening of the hip and knee extensors, proprioceptive re-education, and coordination drills to address persistent asymmetries. To maximise outcomes, rehabilitation intensity, and volume during this window should be increased beyond standard protocols, with close monitoring of functional milestones. Persistent deviations from normative gait profiles highlight the need for extending physiotherapeutic support beyond initial recovery. Literature supports this approach: Bahadori [53] observed that the most significant gains in mobility occurred between week 3 and month 4 post-THR, characterised by sharp improvements in walking velocity, stride length, and cycle time—all responsive to increased rehabilitation intensity. Hodt-Billington et al. [54] highlighted that early improvements in gait symmetry and stance control are crucial for long-term functional outcomes, further reinforcing the importance of targeted training during this phase. Freddolini et al. [55] reported that suboptimal early mobilisation, particularly in assistive devices like crutches, delayed gait restoration, suggesting that intensive and well-structured early rehabilitation accelerates functional recovery. These findings collectively emphasise the neuromuscular adaptability of the early postoperative period and support the implementation of structured, phase-specific training targeting stance control, gait symmetry, and dynamic strength to shorten the recovery timeline and enhance long-term outcomes [56].

Regarding the fifth question, SPM analysis was key in identifying when and where the most significant changes occurred. Unlike traditional point-based comparisons, SPM{F} and SPM{t} analyses provided a high-resolution understanding of the entire gait cycle, uncovering meaningful but subtle biomechanical changes across the pelvic, hip, knee, and ankle joints [57,58]. The pair-wise comparisons clearly showed that these adaptations were not random but clustered around functionally critical periods of the gait cycle, particularly during stance and pre-swing.

To summarise the results, all studied spatio-temporal parameters improved progressively from the first postoperative assessment, even during the early phase between Examination 1 and 2. This period includes the immediate postoperative phase, characterised by surgical wound healing, tissue oedema, pain, and medical instructions to restrict full weight-bearing and limit the hip range of motion. Despite these constraints, measurable functional improvements were achieved. Only after radiological and clinical evaluation confirming implant stability and soft tissue healing were patients permitted to intensify their rehabilitation. The most dynamic improvements in gait were observed between Examinations 2 and 4 (6 weeks to 6 months), coinciding with the period of progressive loading, functional independence, and neuromotor adaptation. However, despite implementing individualised rehabilitation protocols and statistically significant gains at all stages, patients did not reach the normative values achieved by the control group, as is often confirmed in other studies [11]. This highlights the need for early and intensified rehabilitation interventions, particularly between 2 and 3 weeks post-surgery and 3–4 months, to shorten the overall recovery timeline.

Specific movements, particularly hip and knee flexion-extension, demonstrated the highest variability over time, reflecting the functional compensation mechanisms that emerge as patients regain mobility. Colgan et al. [59] observed similar patterns in their sagittal plane gait analysis post-THR, identifying hip and knee flexion-extension as the most variable parameters early after surgery. Their findings confirm that altered hip extensor function often affects knee mechanics, reinforcing the interconnected nature of lower limb biomechanics. This study further underscores the clinical value of restoring hip stability early in rehabilitation to support coordinated joint movement. Additionally, De Pieri et al. [60] applied SPM to evaluate hip contact forces throughout the gait cycle, identifying significant fluctuations modulated by individual patient characteristics during the stance phase. This reinforces the importance of using SPM{t} to detect phase-specific adaptations and supports the argument for personalised rehabilitation strategies. Their work aligns with our findings, showing how SPM enables nuanced tracking of biomechanical evolution, especially in movements with high variability like flexion-extension. Together, these studies validate the role of SPM in revealing functionally relevant gait deviations and shaping targeted clinical interventions. The use of Statistical Parametric Mapping (SPM) allowed for a detailed analysis of continuous gait cycle data, offering a more comprehensive assessment of movement patterns compared to discrete time-point analyses. Applying SPM{t} in post hoc testing provided statistical clarity on the timing and extent of gait adaptations, contributing to a refined understanding of post-THR motor recovery. Clinically, these findings emphasise the importance of targeted rehabilitation interventions tailored to specific recovery phases. Early rehabilitation efforts should prioritise restoring hip stability and weight-bearing control, while mid-to-late-stage interventions should focus on optimising gait symmetry and dynamic balance [59,60].

Gait symmetry, or the lack thereof, post-THR, embodies a nuanced indicator of functional recovery. As noted by Bahadori et al. [61], gait analysis serves as a fundamental tool in evaluating walking ability and long-term physical performance post-THR, underscoring its significance in rehabilitation outcomes. However, the evolution of gait patterns over successive post-operative phases remains inadequately mapped, signalling a gap in current research paradigms.

Identifying specific phases during which significant changes in gait patterns occur post-THR is paramount. Stolarczyk et al. [13] have shown that prosthetic designs, such as larger femoral heads, can influence post-operative gait patterns, hinting at the complexity of factors influencing gait recovery. This emphasises the necessity of identifying critical periods of change to tailor rehabilitation efforts more effectively. By understanding when significant gait improvements or regressions occur, and which movements are most affected, rehabilitation protocols can be customised to address specific deficits. Consequently, this targeted approach can optimise recovery, enhance functional outcomes, and potentially expedite the return to pre-operative activity levels.

Moreover, as discussed by Pagano et al. [12], the utilisation of advanced gait analysis technologies offers promising avenues for capturing detailed gait parameters, facilitating a more nuanced understanding of gait dynamics post-THR. Integrating such technologies in longitudinal studies could illuminate the temporal aspects of gait recovery, offering evidence-based insights to refine and personalise rehabilitation protocols.

One limitation of this study is the absence of muscle activity (EMG) or force plate data, which would provide a more comprehensive understanding of neuromuscular coordination during gait recovery. The inclusion of such data in future studies could enhance the interpretation of joint kinematics and improve the biomechanical profiling of patients following THR. The sample included only female participants, limiting generalisability to broader populations. This resulted from limited access to a female orthopaedic ward and enabled the use of a homogeneous cohort, reducing variability related to sex-specific biomechanics. Future studies should incorporate multi-modal gait assessment and investigate sex-specific responses to rehabilitation following THR. The absence of muscle activity (EMG) and ground reaction force (GRF) data in the present study limits the interpretation of neuromuscular coordination and loading patterns. EMG could elucidate the muscle activation strategies underlying joint kinematics, while GRF analysis would provide insight into asymmetrical loading and compensatory mechanisms. Combined with SPM-based kinematic analysis, these modalities would enable a more comprehensive characterisation of gait recovery and rehabilitation effects. On the other hand, the significance of this study lies in its potential to revolutionise rehabilitation strategies post-THR by advocating for personalised, timing-specific interventions. By leveraging Statistical Parametric Mapping (SPM) to delineate gait pattern differences, this research underscores the necessity for tailored rehabilitation protocols that align with the nuanced phases of post-operative recovery. Consequently, it holds profound implications for biomechanics and clinical practices, enriching the corpus of knowledge on post-THR recovery dynamics. This, in turn, enhances physical activity research by offering granular insights into optimising patient outcomes through bespoke rehabilitative care, thus setting a new precedent for clinical excellence in orthopaedics.

## 5. Conclusions

This study addressed the temporal dynamics and clinical significance of gait pattern changes during rehabilitation following unilateral Total Hip Replacement (THR) in women. The results confirmed that the most significant alterations in spatio-temporal parameters and joint kinematics occurred between 6 weeks and 6 months postoperatively, directly responding to the first and third research questions regarding when and how gait changes evolve. Hip and knee flexion-extension, pelvic tilt, and obliquity were identified as the most variable movements, answering the second question and highlighting key biomechanical adaptations. Although statistically significant improvements were observed across all parameters, patients did not achieve the normative values of the control group, underscoring the clinical importance of early and targeted rehabilitation strategies, supporting insights into the fourth question regarding functional outcomes. Finally, statistical parametric mapping (SPM) enabled the precise identification of phase-specific kinematic changes throughout the gait cycle, validating its utility in detecting subtle yet meaningful gait adaptations in post-THR patients and fulfilling the fifth research objective. These findings provide a robust foundation for refining rehabilitation protocols and support adopting individualised, phase-sensitive approaches to enhance functional recovery.

## Figures and Tables

**Table 1:** Results of the spatio-temporal gait parameters across five examination periods: preoperative (Ex1), 6 weeks (Ex2), 3 months (Ex3), 6 months (Ex4), and 12 months (Ex5) postoperatively. Parameters include velocity, stride length, step length (involved leg), and cycle time. Data are presented as mean ± standard deviation (SD). Statistical significance was assessed using repeated measures ANOVA, with F-values and *p*-values indicating variance and significance levels among periods.

Parameter	Ex1 (Pre-Op)	Ex2 (6 Weeks Post-Op)	Ex3 (3 Months Post-Op)	Ex4 (6 Months Post-Op)	Ex5 (12 Months Post-Op)	F-Value	*p*-Value
Velocity (m/s)	0.42 ± 0.10	0.48 ± 0.08	0.55 ± 0.09	0.62 ± 0.08	0.72 ± 0.06	177.7	0.00
Stride Length (m)	0.85 ± 0.12	0.92 ± 0.10	1.01 ± 0.09	1.08 ± 0.08	1.15 ± 0.07	195.5	0.03
Step Length—Involved Leg (m)	0.32 ± 0.08	0.36 ± 0.07	0.40 ± 0.06	0.44 ± 0.06	0.48 ± 0.05	61.5	0.03
Cycle Time (s)	1.50 ± 0.20	1.40 ± 0.18	1.32 ± 0.15	1.28 ± 0.12	1.22 ± 0.10	141.7	0.01

## Data Availability

The data presented in this study are available from the corresponding author on reasonable request, after anonymisation. The data are not publicly available due to ethical restrictions and the need to protect participant confidentiality, in accordance with the informed consent provided and the data protection regulations of Wroclaw Medical University and Wroclaw University of Health and Sport Sciences. No new data were collected at this stage of the project; all data supporting the findings are contained within the article.

## Abbreviations

The following abbreviations are used in this manuscript:

THR	Total Hip Replacement
SPM	Statistical Parametric Mapping
SPM{F}	SPM F-test continuum
SPM{t}	SPM t-test continuum
Ex1–Ex5	Examination 1 to 5 (time points of gait analysis)
ROM	Range of Motion
